# Association Between Changes in Racial Residential Segregation and Trends in Racial Disparities in Early Mortality in 220 Metropolitan Areas, 2001–2018

**DOI:** 10.1007/s40615-023-01830-z

**Published:** 2023-10-19

**Authors:** Michael Siegel, Madeline Rieders, Hannah Rieders, Leighla Dergham, Rohan Iyer

**Affiliations:** Department of Public Health and Community Medicine, Tufts University School of Medicine, 136 Harrison Avenue, Boston, MA 02111 USA

**Keywords:** Racial health disparities, Structural racism, African Americans, Mortality rates

## Abstract

**Introduction:**

Racial residential segregation has been shown to affect the absolute levels of racial disparities in a wide variety of health outcomes in the USA but it is not known whether changes in segregation also influence these racial health disparities. This study examines the relationship between changes in racial residential segregation over four decades (1980–2020) and trends in racial disparities in early mortality (under age 65) rates among non-Hispanic Black and non-Hispanic White persons across a wide range of health outcomes in 220 metropolitan statistical areas (MSAs) during the period 2001–2018.

**Methods:**

Using the CDC WONDER Underlying Cause of Death database, we derived annual estimates of race-specific death rates and rate ratios for each MSA. We used latent trajectory analysis to examine the relationship between the level of segregation and changes in segregation over time in an MSA and trends in death rate disparities in that MSA.

**Results:**

The trajectory analysis resulted in a linear, three group model in which trajectory Groups 1 and 2 had decreasing trends in the ratios of Black to White death rates over time while in Group 3, the disparity remained almost constant over time. Increases in the level of segregation in an MSA from 1980 to 2000 were significantly associated with the likelihood that the MSA was in Group 3 and experienced no improvement in racial health disparities in mortality over time.

**Conclusion:**

This paper provides new evidence that changes in segregation are related to trends in racial health disparities in mortality rates over time.

**Supplementary Information:**

The online version contains supplementary material available at 10.1007/s40615-023-01830-z.

## Introduction

Structural racism has been shown to affect the absolute levels of racial disparities in a wide variety of health outcomes in the USA [1–4]. One aspect of structural racism that has received much attention is racial residential segregation [5–7]. There have been many studies demonstrating a cross-sectional relationship between racial segregation and racial health disparities at the neighborhood [8], Census tract [9], county [10, 11], state [12–14], or metropolitan area [15–17] level at a single point in time. However, it is also critical to understand whether and how both racial segregation and racial health disparities are changing over time and how these changes relate to each other.

## Racial Residential Segregation

Racial residential segregation is defined as “the physical separation of the races in residential contexts” [5, p. 404]. It is “an institutional mechanism of racism that was designed to protect whites from social interaction with blacks” [5, p. 404]. As explained by Williams and Collins, racial residential segregation “was imposed by legislation, supported by major economic institutions, enshrined in the housing policies of the federal government, enforced by the judicial system, and legitimized by the ideology of white supremacy that was advocated by churches and other cultural institutions” [5, p. 405]. The consequences of residential segregation include lower socioeconomic status for the segregated group due to limited access to employment and education, lower expenditures for education and public services, higher exposure to environmental pollutants, and a less health physical, social, and built environment [5–10].

## Trends in Racial Residential Segregation

A number of papers have explored changes in the level of segregation in US cities and metropolitan areas over time. For example, Fischer et al. analyzed trends in racial segregation across US metropolitan areas during the period 1960–2000 [18]. Hess examined trends in Black-White residential segregation in metropolitan areas from 1980 to 2018 [19]. Fischer presented trends in racial segregation at the city and suburban levels between 1980 and 2000 [20]. Massey and Tannen characterized the role of suburbanization in altering levels of racial segregation across US metropolitan areas during the period 1970–2010 [21]. Reardon et al. examined changes in the geographic scale of racial residential segregation in metropolitan areas between 1990 and 2000 [22]. Lichter et al. estimated trends in three measures of segregation across 300 metropolitan statistical areas from 1990 to 2020 [23]. However, none of these studies examined the relationship between these observed trends in segregation and changes in racial health disparities.

## Trends in Racial Health Disparities

Another set of studies have examined trends in racial health disparities over time at the county, state, and national levels. For example, the National Center for Health Statistics presented a comprehensive review of nationwide trends in a wide variety of racial/ethnic health disparities over the 20-year period 2000–2020 [24]. Mahajan et al. reported trends in racial disparities in health status and health care access at the national level during the period 1999–2018 [25]. Goldfarb et al. presented trends in racial disparities in low birthweight rates at the county level for the period 2003–2013 [26]. Speights et al. investigated state-level trends in reducing the Black-White infant mortality gap between 1999 and 2013 [27]. However, none of these studies examined the potential association between changes in racial segregation and the observed trends in racial health disparities.

## A Critical Gap in the Literature

Few studies have examined the relationship between *changes* in racial segregation and *changes* in racial health disparities over time. This is critical because the purpose of studying the relationship between segregation and racial health disparities is not to simply understand these disparities but to identify ways to reduce or eliminate them. Therefore, we need to know not only whether segregation and racial health disparities are present at a given point in time but whether reducing segregation will in turn reduce racial health disparities over time. Thus, the primary aim of this study is to assess whether changes in racial residential segregation in metropolitan areas are associated with subsequent changes in the trajectories over time in the magnitude of racial health disparities across a wide variety of health outcomes.

We are aware of two studies that related baseline levels of racial segregation to subsequent trends in racial disparities, one studying firearm homicide at the city level [28]; the other studying COVID infection rates at the city level [29]. Neither of these studies measured changes in segregation.

In fact, we are aware of only two studies that have related changes in racial segregation to changes in racial health disparities. Both of these were conducted at the individual level. Kershaw et al. conducted a national prospective study of within-person changes in blood pressure over a 25-year period, finding that people who moved to less segregated areas experienced significant reductions in blood pressure [30]. Wang et al. reported that children who moved out of highly segregated neighborhoods experienced significant improvement in their self-rated health over time [31].

Our study adds to the existing literature by examining the relationship between changes in racial residential segregation and trends in racial disparities in mortality across a wide range of health outcomes in 220 metropolitan statistical areas (MSAs) during the period 2001–2018. We examine the relationship between both baseline levels of segregation and trajectories of racial mortality disparities and between changes in segregation over time and these trajectories. To our knowledge, this is the first study to examine the association between changes in racial segregation and changes in racial health disparities in mortality at a young age (less than age 65) for a wide range of diseases.

## Methods

### Geographical Level of Analysis

We chose to conduct this analysis at the MSA level because as Kotecki et al. explain: “The level of geography was chosen because it is well suited to reflect the housing and labor markets that are responsible for creating patterns of segregation [15, pp. 150–151].”

## Measures and Data Sources

### Death Rates and Death Rate Ratios

Using the CDC WONDER Underlying Cause of Death database [32], which covers the entire USA, we obtained annual, county-specific counts of deaths and populations for the Black, non-Hispanic, and White, non-Hispanic subpopulations, which we added across counties to derive estimates for each of the 220 MSAs in our sample. We included 12 causes of death: HIV (ICD-10 codes B20-B24), colon cancer (C18), lung cancer (C34), breast cancer (C50), prostate cancer (C61), diabetes (E10-E14), hypertension (I10-I15), cerebrovascular disease (I60-I69), respiratory disease (J00-J98), genitourinary disease (N00-N98), pregnancy (O00-O99), and firearm homicide (X93-X95, Y22-Y24, Y35.0). We calculated death rates only for those under age 65 because racial disparities tend to disappear as White people reach high levels of longevity and then eventually develop some of the diseases that Black people tend to develop at much earlier ages. For example, in 2020, the all-ages White death rate from cerebrovascular disease (58.2 per 100,000) was actually higher than that for Black people (51.4 per 100,000) [32]. However, examining only deaths of younger people below age 65 tells a different story. Here, there was nearly a twofold disparity, with Black people having a death rate of 15.4 per 100,000 compared to 7.9 per 100,000 among White people [32]. Overall death rates were calculated by combining death rates from each of the 12 causes listed above.

In order to protect privacy, CDC does not report death counts of less than 10. In order to avoid having any missing data, we combined 5 years of data at a time, deriving 5-year moving averages of death rates. For example, the data point for 2001 was derived from the combined death counts for the 5-year period 1999–2003. The data point for 2018 was derived from the combined death counts for the 5-year period 2016–2020. This procedure tends to “smooth” the curve of the racial disparity trends over time. This is appropriate for phenomena that are not expected to change very quickly, such as death rates. It would be less appropriate to use for phenomena that change very quickly from year to year, such as gas prices. We do not believe that this procedure led to a misinterpretation of the trends in racial disparities because death rates tend to reflect long-term characteristics of a population. One exception might be an event such as the COVID-19 pandemic; however, our data estimates do not go past the year 2018.

Death rates were calculated by dividing the number of deaths by the total population of a given group. Death rate ratios were calculated by dividing the Black death rate by the White death rate. These annual death rate ratios served as the outcome variable of our analyses.

### Measures of Racial Residential Segregation

Using data from the Decennial Censuses [33], we calculated two common measures of Black-White racial segregation at the Census tract level for each MSA for the years 1980 [34], 1990 [35], 2000 [33], 2010 [33], and 2020 [33]. To ensure that our findings were robust, we used two different common measures of racial segregation: the index of dissimilarity and the entropy index. Both were calculated at the Census tract level. The Decennial Census data for 2000, 2010, and 2020 were downloaded directly from the U.S. Census Bureau website [33]. The data for 1980 [34] and 1990 [35] were obtained from the Inter-university Consortium for Political and Social Research (ICPSR).

#### Index of Dissimilarity

The index of dissimilarity, one of the most commonly used indicators of racial segregation, measures the proportion of people of one racial group that would have to move from a local level of geography in order to integrate a larger level of geography [7]. The scale runs from 0 to 100, with 0 indicating perfect integration and 100 indicating complete segregation. We calculated the index of dissimilarity for the non-Hispanic White and non-Hispanic Black populations using the Census tract as the lower level of geography and the MSA as the larger unit. The formula was:

$${\text{I}}=\frac{1}{2}\sum \limits_{t=1}^{n}\left|\left({B}_{t}/{B}_{\text{MSA}}\right)-\left({W}_{t}/{W}_{\text{MSA}}\right)\right|$$

where ${B}_{t}$ is the number of Black people in Census tract *t*, ${B}_{\text{MSA}}$ is the number of Black people in the MSA as a whole, ${W}_{t}$ is the number of White people in Census tract *t*, ${W}_{\text{MSA}}$ is the number of White people in the MSA as a whole, and the sum is across all $n$ Census tracts within the MSA.

#### Entropy Index

Entropy is a measure of the proportion of racial groups (in this case, Black and White) in a lower geographical unit (in this case, the Census tract) in comparison to each group’s expected proportion in that unit based on a weighted average of each racial group’s presence in all units within the larger geographic unit (in this case, the MSA) [36–38]. The entropy index runs from 0, representing perfect integration to 100, representing complete segregation.

For a given Census tract with $n$ racial groups, its entropy (*E*_*t*_) is defined as:

$${E}_{t}=\sum \limits_{r\text{=}1}^{n}{P}_{rt}\left(1/{P}_{rt}\right) \, ,$$

where ${P}_{rt}$ is the proportion of tract $t$’s population comprised of racial group $r$.

The entropy for an MSA with $n$ racial groups is defined similarly:

$$\sum \limits_{r\text{=}1}^{n}{P}_{r{\text{MSA}}}\left(1/{P}_{r{\text{MSA}}}\right),$$

where ${P}_{r{\text{msa}}}$ is the proportion of the MSA’s population comprised of racial group $r$.

The entropy index (EI) is then defined as:

$${\text{EI}}=\sum \limits_{1}^{t}\frac{{\text{PO}}{\text{P}}_{t}\left({E}_{\text{MSA}}-{E}_{t}\right)}{{{\text{PO}}{\text{P}}_{\text{MSA}}\left({E}_{\text{MSA}}\right)}},$$

where ${\text{PO}}{\text{P}}_{t}$ is the population of tract $t$ and ${\text{PO}}{\text{P}}_{\text{MSA}}$ is the population of the MSA, and the summing takes places across each Census tract in the MSA.

## Selection of Metropolitan Statistical Areas

Because our aim was to assess changes in racial segregation over a 40-year period (1980–2020), it was critical that we select a consistent set of metropolitan areas with the identical county composition over the entire study period. However, the Office of Management and Budget, which designates metropolitan statistical areas, has changed its delineations of MSA in 1980, 1990, 2000, and 2010, adding and deleting MSAs and adding or deleting counties from MSAs [39]. For example, in 1980, there were 315 MSAs (then called standard metropolitan statistical areas), which contained 168.9 million people among 725 counties. In 2020, there were 404 MSAs comprising 286.1 million people across 1181 counties. A second goal in choosing MSAs was to ensure that each MSA and each county included in the sample have a large enough population—and particularly, a large enough Black population—to derive stable estimates of mortality rates throughout the study period. To identify a consistent sample of MSAs, defined in a way that would allow stable race-specific death rate estimates, we began with the 2020 MSAs and followed them back to 1980, obtaining the total, Black, and White populations of each MSA as well as each county within each MSA. We settled on the following inclusion criteria: (1) MSA must have more than 100,000 total population in 1980; (2) MSA must have greater than 5000 Black population in 1980; (3) MSA must not be missing Census tract population estimates for 1980; (4) each county within the MSA must have at least 3000 Black population in 1980; (5) each county must comprise at least 10% of the total MSA population. Our resulting sample consisted of 220 MSAs comprising a total of 353 counties, defined consistently throughout the period 1980–2020 (Appendix Table 1).

## Data Analysis

### Latent Trajectory Analysis

We examined the relationship between the degree of racial segregation and trends in death rate disparities. We used latent trajectory analysis to identify groups of MSAs sharing similar trajectories in racial disparity trends during the study period and to determine whether the level of segregation in an MSA was significantly related to the likelihood that it was classified in one trajectory group over another. We then investigated whether changes in segregation levels over time were significantly related to the likelihood that an MSA followed one trajectory over another. In this way, we were able to assess the relationship between both the absolute level of segregation and changes in segregation on both baseline levels and trends in racial health disparities.

Latent trajectory analysis is a trend modeling technique that enables one to identify groups of units (in this case, MSAs) that follow similar trajectories over time [40–45]. There are two basic components of a trajectory: (1) a baseline level at the start of the period (i.e., the intercept) and (2) a trend across the period. The trend may be linear, quadratic, cubic, or higher order, and there may be two, three, four, or more latent groups. These components of the trajectories are determined primarily by examining how closely the models fit the observed data. Specifically, the trajectory groups are formed by “identifying distinctive clusters of individual trajectories within the population…” [42, p. 3]. The best classification for each individual trajectory is determined using maximum likelihood to minimize model error [42]. The procedure uses a search routine “to locate empirically the parameter estimates that maximize the likelihood function” [40, p. 37]. Latent trajectory analysis has been used in a wide variety of public health areas to gain a better understanding of trends in a variety of health outcomes, including racial violence [46], crime rates [47], city-level homicide rates [48], and racial disparities in firearm homicide across cities [28].

We conducted the latent trajectory analysis using the *traj* procedure for STATA, which was developed and made publicly available by Jones and Nagin [40–45]. The first goal of the analysis was to identify the number of and patterns of distinct trajectories of the Black-White disparity in death rates from 2001 to 2018 and to classify each MSA into one of these trajectory groups, thus creating clusters of MSAs with similar trajectories of racial disparities over time. The second goal of the analysis was to determine whether (1) baseline levels of racial segregation and/or (2) changes over time in racial segregation were significant predictors of the likelihood that a particular MSA was in one trajectory group compared to others. If the racial disparities in health outcomes across MSAs are not impacted by racial residential segregation, then there should be no relationship between what trajectory group an MSA is in and its level of racial segregation. Thus, this analysis tests the proposition that an MSA’s trajectory group is indeed influenced by its level of racial segregation. The null hypothesis is that trajectory group membership is unrelated to either baseline levels of segregation or trends in segregation over time. Under the null hypothesis, the coefficients for both of these variables should be zero.

To identify the trajectory groups, the outcome variable was the annual racial disparity in death rates (defined as the ratio of the Black, non-Hispanic death rate to the White, non-Hispanic death rate). The time variable was the year 2001–2018. Since the death rate ratios were normally distributed, we used a censored normal distribution for the outcome variable. In deciding how many latent groups of MSAs to model, we were guided by the dual aims of first, not missing clusters with trajectories that were distinct from those of others and second, not using so many groups such as to generate low-fit models, as evidenced by the AIC and BIC statistics. In deciding the order for the trends (i.e., linear, quadratic, and cubic), we were guided by the dual aims of first, not overinterpreting small fluctuations from year-to-year (as racial disparities in death rates would not be expected to change very quickly over time) and second, ensuring tight model fit both visually and by examination of the AIC and BIC fit statistics.

After settling on the most appropriate trajectory model, we then evaluated whether baseline levels of racial segregation (the index of dissimilarity or entropy index in an MSA in 1980) were associated with latent trajectory group membership. Separately, we evaluated whether the change in levels of racial segregation from 1980 to 2000 were associated with subsequent trajectories during the 2001–2018 period. Finally, we included both baseline segregation (1980) and change in segregation (1980 to 2000) as time-constant variables in the same model in order to determine whether, after controlling for baseline values of racial segregation, the change in racial segregation was significantly associated with trajectory group membership.

The reason we chose to look at changes in segregation between 1980 and 2000 and changes in racial disparities two decades later (2001–2018) is because we hypothesized that it would take on the order of 20 years before changes in segregation resulted in measurable differences in chronic disease mortality rates.

To ease the interpretation of odds ratios, we scaled the variables so that the reported odds ratios indicate the increase in odds of membership in one trajectory group versus the referent group for each increase of 5 points in the index of segregation or entropy index or for each conducted using STATA version 17 (College Station, TX: StataCorp) with the *traj* plug-in [42].

## Results

### Descriptive Results

**Fig. 1:**
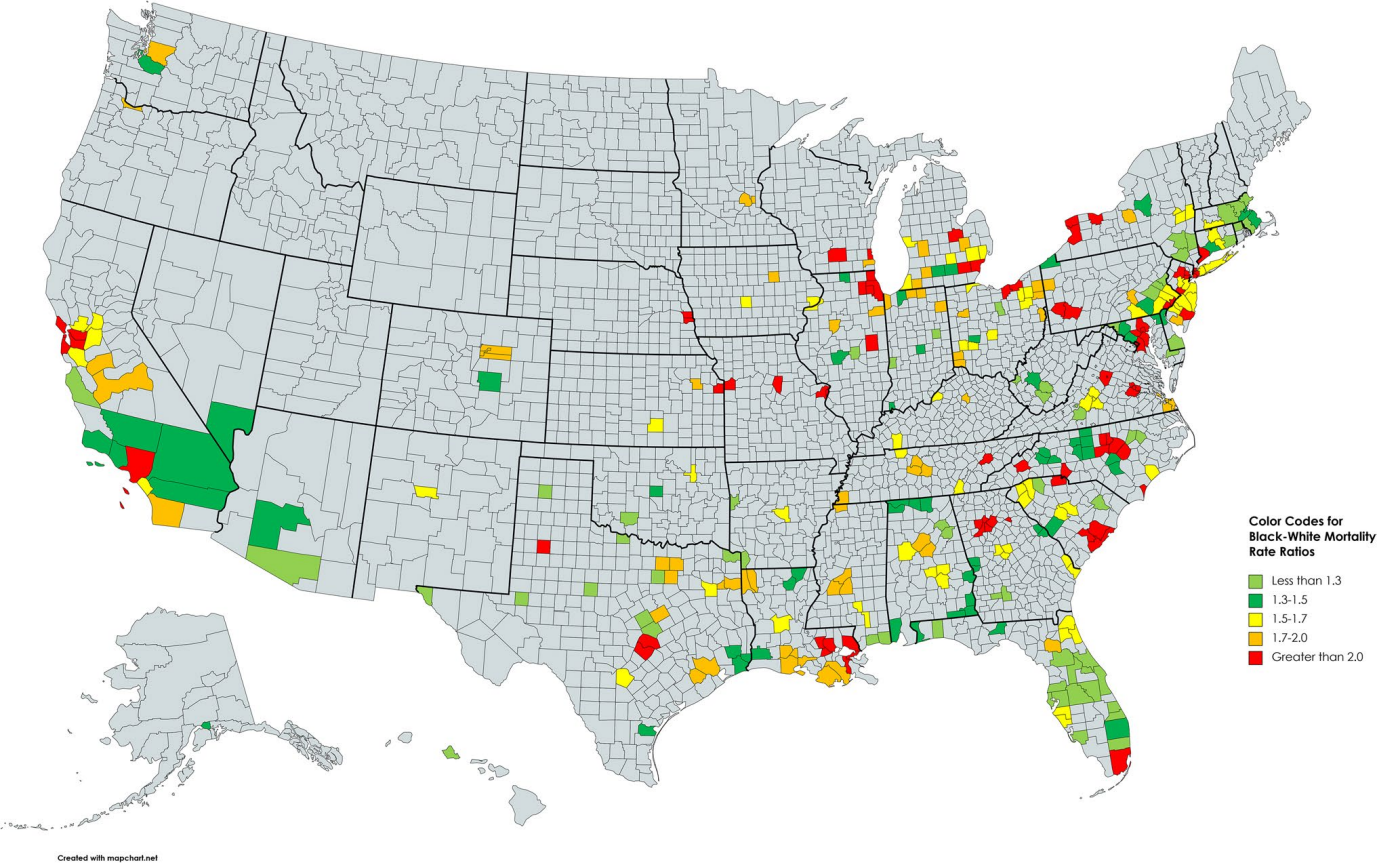
Heat map showing the Black-White mortality rate ratio for 220 metropolitan statistical areas for selected underlying causes of death in people under age 65, 2016–2020 average

**Fig. 2:**
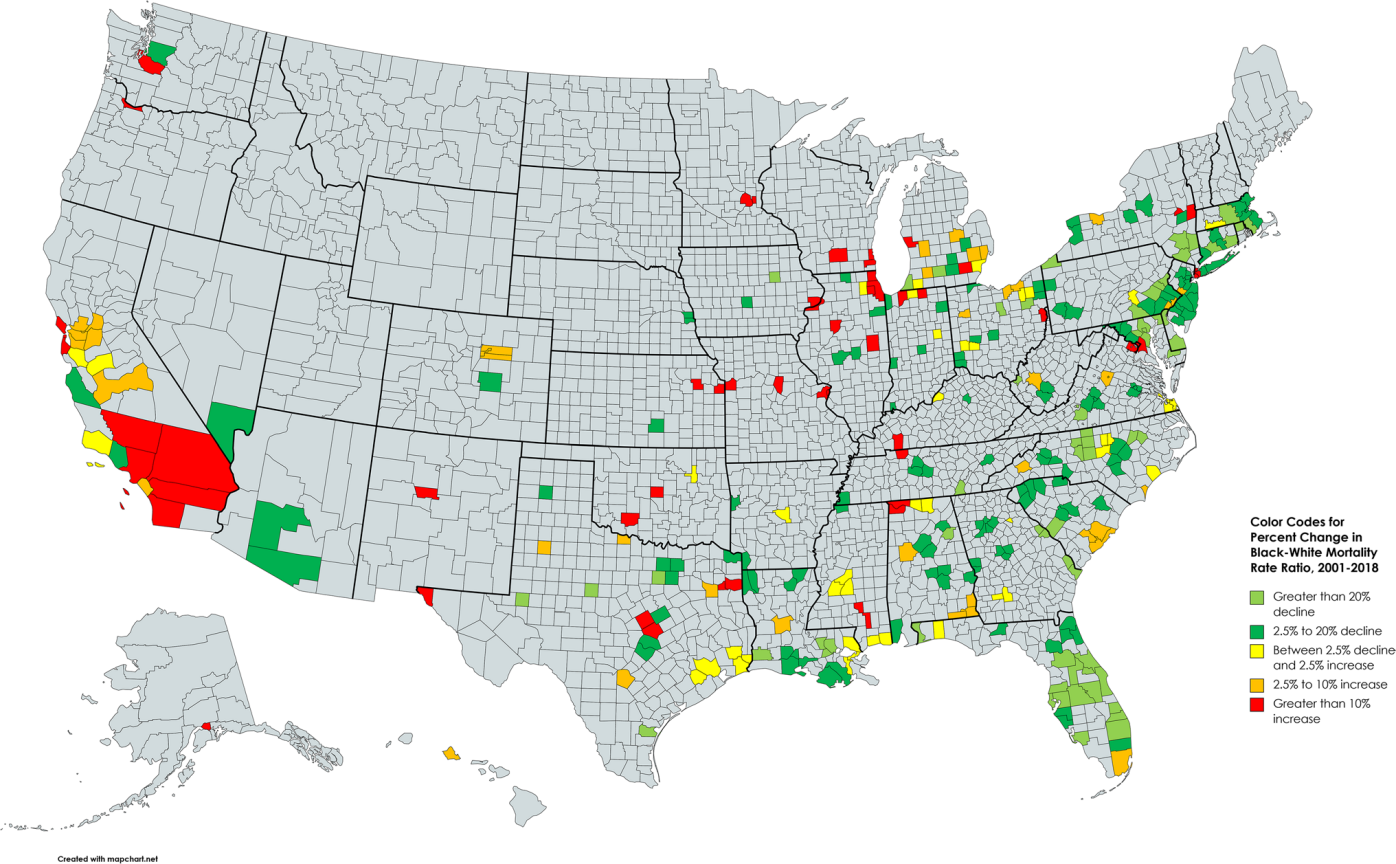
Heat map showing the percentage change in the Black-White mortality rate ratio from 2001 to 2018 for selected underlying causes of death in people under age 65—220 Metropolitan Statical Areas

During the period 2001–2018, 215 of the 220 MSAs had an average Black-White mortality rate ratio greater than one, indicating a racial disparity in death rates for the selected diseases among people below the age of 65 (Appendix Table 1). The average racial disparity in death rates across all 220 MSAs during the study period was 1.67. The average mortality rate ratios ranged from a low of 0.74 in Honolulu [HI] to a high of 4.07 in San Jose-Sunnyvale [CA]. The five MSAs with the lowest average death rate ratios were Honolulu [HI] (0.74), Hagerstown-Martinsburg [MD/WV] (0.86), Amarillo [TX] (0.93), Ocala [FL] (0.95), and Elkhart-Goshen [IN] (0.99). The five MSAs with the highest average death rate ratios were San Jose-Sunnyvale [CA] (4.07), Washington-Arlington [DC/VA] (3.35), Athens-Clarke County [GA] (3.19), New Brunswick-Lakewood [NJ] (2.12), and Chicago-Naperville-Evanston [IL] (2.87). Figure 1 is a heat map displaying the magnitude of the Black-White death rate ratio for each MSA in the year 2018. Figure 2 displays the percentage change in the Black-White death rate ratio for each MSA over the period 2001–2018.

The baseline level of segregation in 1980, as measured by the index of dissimilarity, ranged from a low of 0.34 in Jacksonville [NC] to a high of 0.90 in Chicago-Naperville-Evanston [IL] (Appendix Table 1). The MSAs with the lowest level of segregation at baseline were Jacksonville [NC] (0.34), Fayetteville [NC] (0.39), Anchorage [AK] (0.39), Salisbury [MD/DE] (0.39), and Lawton [OK] (0.40). The MSAs with the highest level of segregation at baseline were Chicago-Naperville-Evanston [IL] (0.90), Gary [IN] (0.89), Cape Coral-Fort Myers [FL] (0.89), Cleveland-Elyria [OH] (0.85), and North Port-Sarasota-Bradenton [FL] (0.85). The change in the level of segregation from 1980 to 2000, measured by the index of dissimilarity, ranged from a decrease of 0.38 in Elkhart-Goshen [IN] to an increase of 0.17 in Salisbury [MD/DE]. The five MSAs with the largest decline in segregation from 1980 to 2000 were Elkhart-Goshen [IN] (− 0.38), Odessa [TX] (− 0.30), Port St. Lucie [FL] (− 0.29), Springfield [IL] (− 0.28), and San Antonio-New Braunfels [TX] (− 0.26). The five MSAs with the largest increase in segregation from 1980 to 2000 were Salisbury [MD/DE] (+ 0.17), New Brunswick-Lakewood [NJ] (+ 0.14), San Jose-Sunnyvale [CA] (+ 0.13), Santa Maria-Santa Barbara [CA] (+ 0.12), and Spartanburg [SC] (+ 0.12).

### Trajectory Analysis Results

**Fig. 3:**
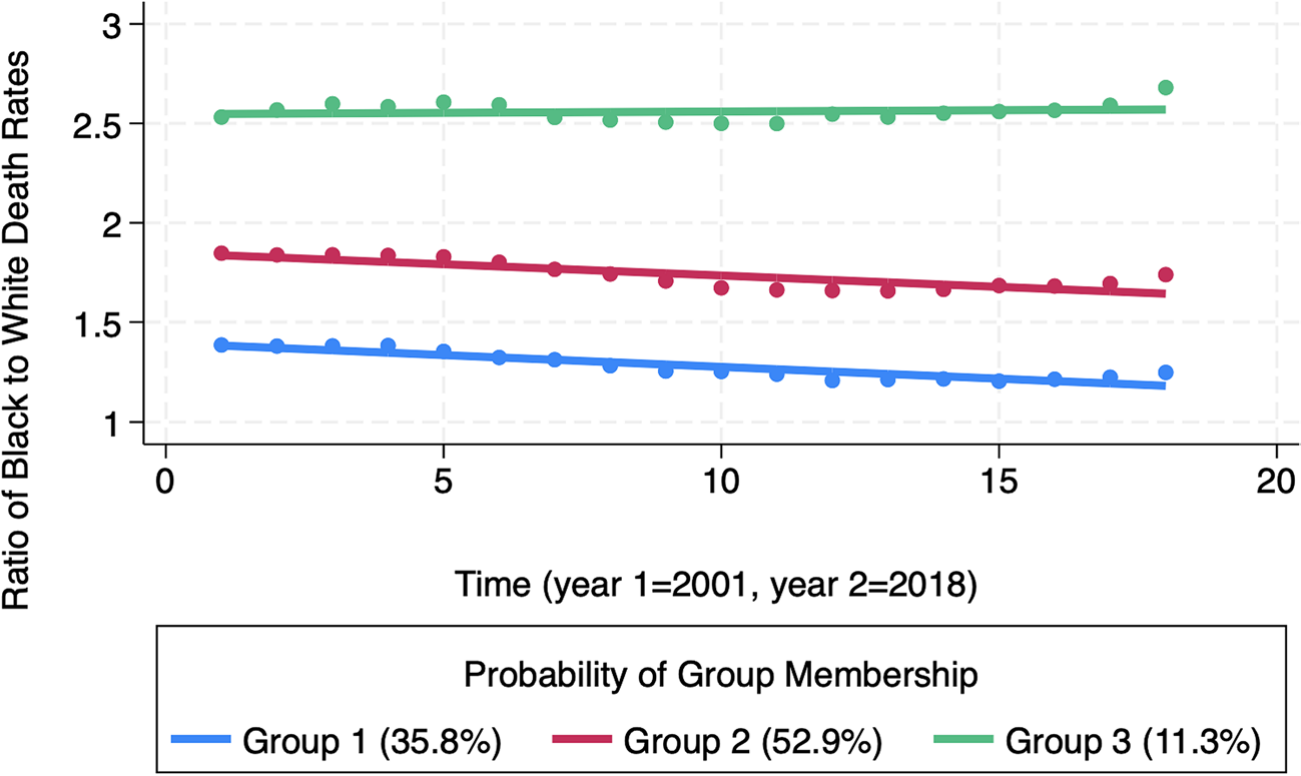
Latent trajectory model of trends in the ratio of Black to White death rates, 2001–2018

Based on the model fit parameters, we found that a linear, three group model was the best fit to describe the trends in the racial disparities in death rates from 2001 to 2018. Trajectory groups 1 and 2, which had the highest MSA group membership, had decreasing trends in the ratios of Black to White death rates over time (Fig. 3). Group 3 had the lowest MSA group membership, started with the highest baseline racial disparity in death rates in 2001, and the disparity remained almost constant over time. Despite very similar decreasing trends in racial disparities in death rates, Group 1 can be distinguished from Group 2 due to having the lowest baseline ratio of Black to White death rates in 2001.

Table 1 displays a descriptive statistics profile of each of the three trajectory groups, where presented values are the average of all values from MSAs within the group. Baseline segregation in column two is displayed as each group’s index of dissimilarity in 1980, on a scale of 0 to 100. Segregation progressively worsened by group, with Group 3 possessing the highest degree of segregation at baseline. Likewise, changes in segregation, as assessed by changes in the index of dissimilarity between baseline of 1980 and 2020, are displayed by trajectory group in the third column. On average, MSAs in each group did see a decline in segregation over the time period, with MSAs in Group 1 having the greatest progress and Group 3 having the least*.* As displayed in column four, the baseline racial disparity in death rates was progressively higher moving from Group 1 to Group 2 to Group 3. In addition to Group 3 having the worst disparity at baseline in 2001, it was the only group to experience an increase in the disparity over time.

**Table 1:** Descriptive statistics profile of each trajectory group

Trajectory group (%)	Average baseline segregation (1980)	Average change in segregation (1980–2020)	Average baseline Black-White mortality rate ratio (2001)	Average change in Black-White mortality rate ratio (2001–2018)
1 (35.8%)	61.4	− 15.9	1.3	− 7.4%
2 (52.9%)	66.3	− 13.8	1.7	− 5.1%
3 (11.3%)	72.4	− 11.4	2.6	+ 6.1%

Table 2 displays the results of latent trajectory analysis for all three groups and the effect of both the baseline index of dissimilarity (1980) and change in the index of dissimilarity (1980–2000) as time-constant variables on the likelihood of MSA group membership, using both Group 1 and Group 2 as reference groups. We included both of these covariates in order to determine whether a change in the index of dissimilarity would still be significantly associated with trajectory group membership even after controlling for the baseline values of the index of dissimilarity. The odds ratios indicate how much more likely an MSA was to be in a specific trajectory group compared to the reference group as the index of dissimilarity of an MSA increased by five points. Thus, the odds ratio of 1.42 for Group 2, using Group 1 as the reference group, indicates that for every five point increase in the baseline index of dissimilarity in 1980 for an MSA, the MSA was 1.42 times more likely to be in Group 2 than Group 1 (*p* < 0.001). From the same analysis, for each five point increase in the baseline index of dissimilarity in 1980, an MSA was 2.10 times more likely to be in Group 3 than Group 1 (p < 0.001). When using Group 2 as the reference group, for each five point increase in the baseline index of dissimilarity, an MSA was 1.47 times more likely to be in Group 3 than Group 2 (p = 0.002). Thus, higher levels of segregation in an MSA at baseline were associated with steadily increasing odds of being in Group 2 or Group 3.

**Table 2:** Results of latent trajectory analysis and the effect of racial segregation (baseline 1980 and change 1980–2000) on likelihood of MSA group membership using index of dissimilarity as the measure of racial residential segregation

Group	Parameter	Estimate	Standard error	*p* value	
Group 1	Intercept	1.398	0.016	< 0.001	
	Linear	− 0.012	0.001	< 0.001	
Group 2	Intercept	1.851	0.013	< 0.001	
	Linear	− 0.011	0.001	< 0.001	
Group 3	Intercept	2.546	0.026	< 0.001	
	Linear	0.001	0.002	0.58	
Effect of racial segregation on likelihood of MSA group membership (time-constant)		Estimate	Standard error	*p* value	Odds ratio
Group 1	Reference group				
Group 2	Index of dissimilarity 1980	0.352	0.082	< 0.001	1.42
	Change in index of dissimilarity 1980–2000	0.565	0.135	< 0.001	1.76
Group 3	Index of dissimilarity 1980	0.740	0.141	< 0.001	2.10
	Change in index of dissimilarity 1980–2000	1.058	0.216	< 0.001	2.88
Effect of racial segregation on likelihood of MSA group membership (time-constant)		Estimate	Standard error	*p* value	Odds ratio
Group 2	Reference group				
Group 3	Index of dissimilarity 1980	0.388	0.124	0.002	1.47
	Change in index of dissimilarity 1980–2000	0.493	0.185	0.008	1.64

Even after controlling for the baseline level of segregation in 1980, the analyses showed that *changes* in the level of segregation in an MSA from 1980 to 2000 were significantly associated with the likelihood that the MSA was in a particular trajectory group, with greater increases in segregation being associated with steadily increasing odds of an MSA being in a higher trajectory group (Table 2). For each increase of five points in the change in the index of dissimilarity from 1980 to 2000, an MSA was 1.76 times more likely to be in Group 2 than Group 1 (*p* < 0.001) and 2.88 times more likely to be in Group 3 than Group 1 (*p* < 0.001). Using Group 2 as the referent group, for each increase of five points in the change in the index of dissimilarity from 1980 to 2000, an MSA was 1.64 times more likely to be in Group 3 than Group 2 (*p* = 0.008).

Table 3 displays the same relationships as in Table 2, except using the entropy index as the measure of racial residential segregation. The results are essentially unchanged. For each five point increase in the baseline entropy index, an MSA was 1.63 times more likely to be in Group 2 than Group 1 (*p* < 0.001) and 2.37 times more likely to be in Group 3 than Group 1 (*p* < 0.001). When using Group 2 as the reference group, for each five point increase in the baseline entropy index, an MSA was 1.45 times more likely to be in Group 3 than Group 2 (*p* < 0.001).

**Table 3:** Results of latent trajectory analysis and the effect of racial segregation (baseline 1980 and change 1980–2000) on likelihood of MSA group membership using entropy index as the measure of racial residential segregation

Group	Parameter	Estimate	Standard error	*p* value	
Group 1	Intercept	1.399	0.017	< 0.001	
	Linear	− 0.012	0.001	< 0.001	
Group 2	Intercept	1.852	0.013	< 0.001	
	Linear	− 0.011	0.001	< 0.001	
Group 3	Intercept	2.555	0.026	< 0.001	
	Linear	0.001	0.002	0.58	
Effect racial segregation on likelihood of MSA group membership (time constant)		Estimate	Standard error	*p* value	Odds ratio
Group 1	Reference group				
Group 2	Entropy index 1980	0.488	0.103	< 0.001	1.63
	Change in entropy index 1980–2000	0.697	0.183	< 0.001	2.01
Group 3	Entropy index 1980	0.861	0.140	< 0.001	2.37
	Change in entropy index 1980–2000	1.189	0.279	< 0.001	3.28
Effect racial segregation on likelihood of MSA group membership (time constant)		Estimate	Standard error	*p* value	Odds ratio
Group 2	Reference group				
Group 3	Entropy index 1980	0.373	0.10565	< 0.001	1.45
	Change in entropy index 1980–2000	0.492	0.23524	0.037	1.63

After controlling for the baseline entropy index in 1980, the analyses showed that once again, *changes* in the level of segregation in an MSA from 1980 to 2000 were significantly associated with the likelihood that the MSA was in a particular trajectory group, with greater increases in segregation being associated with steadily increasing odds of an MSA being in a higher trajectory group (Table 3). For each increase of five points in the change in the entropy index from 1980 to 2000, an MSA was 2.01 times more likely to be in Group 2 than Group 1 (*p* < 0.001) and 3.28 times more likely to be in Group 3 than Group 1 (*p* < 0.001). Using Group 2 as the referent group, for each increase of five points in the change in the entropy index 1980 to 2000, an MSA was 1.63 times more likely to be in Group 3 than Group 2 (*p* = 0.037).

Table 4 displays the estimated years that the disparity in Black-to-White death rates would end in each of the three trajectory groups, based on the trend lines for each group. Because Groups 1 and 2 contain MSA groups that have seen slow progress, as defined by a small decrease in the disparity between the baseline of 2001 and 2018, these groups are projected to see the end of racial disparities in death by 2034 and 2075, respectively. In contrast, Group 3, which had the highest disparity ratio at baseline, contained MSAs that did not see progress in the disparity during the period of assessment. With a positive slope in this trajectory group’s trendline, the racial disparities are only expected to worsen in the coming years.

**Table 4:** Prediction of the end of the Black-to-White death disparity by trajectory groups

Trajectory group	Estimated year of the end of the disparity
1	2034
2	2075
3	-

To examine whether the observed relationship between racial segregation and racial disparities in mortality were attributable to the general economic status of an MSA, rather than to racial segregation, we repeated the analyses including a measure of economic status: the percentage of residents in an MSA who rented, rather than owned their home. The results were essentially unchanged, whether we used rental housing data for 1980 or for 2000.

The trajectory groups to which each MSA was assigned are shown in Appendix Table 1.

## Discussion

To the best of our knowledge, this is the first study to examine the association between changes in segregation over time on trends in racial disparities in mortality rates between non-Hispanic Black and non-Hispanic White people. We examined trends in death rates between 2001 and 2018 among people under age 65 in 220 metropolitan statistical areas for a wide range of diseases with known racial disparities between these groups. We found that both higher baseline levels of racial segregation and lack of progress in reducing segregation over time were associated with higher levels of racial disparities in mortality and a lower likelihood of reducing these disparities. Even after controlling for the baseline level of segregation in an MSA in 1980, each five-point increase in the change in the index of dissimilarity in an MSA from 1980 to 2000 was associated with a near tripling of the likelihood that the MSA was in a trajectory group characterized by both high levels of racial disparities in mortality rates in 2001 and by a failure to reduce these disparities between 2001 and 2018.

Our findings build upon those of two longitudinal studies showing that individuals moving to more segregated areas experience increases in blood pressure [30] and declines in self-rated health [31]. A limitation of these previous studies is that people who are able to move out of segregated areas may be more affluent and therefore more likely to experience positive health outcomes, creating a selection bias. Conducting the analysis at the MSA level may avoid selection bias that may be present in the individual-level studies. The individual-level studies found that people who moved to less segregated areas experienced better health. However, there is a major potential confounding variable: socioeconomic status. It is possible that people who can afford to move to less segregated areas are wealthier than those who cannot and it is possible that differences in socioeconomic position, rather than segregation, could explain the observed changes in health status. As Kotecki et al. explain, conducting the analysis at the MSA level may “minimize selection bias, as forces operating to sort people into neighborhoods are weaker between metropolitan areas as opposed to within metropolitan areas [15, pp. 150–151].” Here, we study large metropolitan areas which reduces selection bias because individual migration is unlikely to affect the overall death rate in a large geographical area. Thus, this study may help address the issue of selection bias in the two previous individual-level studies.

Two particular aspects of our results add validity to the finding that changes in racial residential segregation are associated with levels of and trends in racial disparities in mortality. First, even when the statistical model was not “told” what the baseline level of segregation was in each MSA, changes in segregation alone were predictive of which trajectory group an MSA was most likely to fall under. Furthermore, when the model was “told” the baseline level of segregation, changes in segregation were still a significant predictor of trajectory membership. Thus, it appears that baseline levels of segregation and trends in segregation are independently related to racial disparities in mortality.

Another strength of this study is that we examined two different measures of racial residential segregation: the index of dissimilarity and the entropy index. There was little to no difference between the results using one index or the other. There was also no change in the results when we controlled for the overall economic status of an MSA—both in 1980 and 2000—as measured by the percentage of the MSA’s residents who rented rather than owned housing. Thus, the observed findings do not appear to be explained by differences in overall levels of disadvantage across the MSAs.

The difference between the MSA trajectory groups is worth noting. Based on existing trends in racial disparities in mortality, Group 1 is on track to eliminate its disparity by 2034. However, Group 2 is not predicted to eliminate its disparity until 2075, and Group 3 is not making any progress and is not on track to reduce, much less eliminate its disparities unless major changes are made.

Several policies and programs to reduce racial residential segregation have been suggested and/or attempted, including providing housing vouchers to Black individuals and families, banning discrimination by landlords on the basis of income, housing mobility programs, fair market rent policies, housing search counseling, eliminating exclusionary zoning practices, instituting inclusionary zoning, and public housing redevelopment [49]. In addition, school desegregation, if performed across large areas and combined with changes in land use policy, can be effective in at least countering many of the adverse consequences of residential segregation, if not actually promoting residential integration [10]. Johnson provides examples of success stories, demonstrating that desegregation leads not only to improvement in education, job opportunities, and economic status but also to improvements in health [50]. One of the success stories was Charlotte, North Carolina, in which school desegregation promoted residential integration, but sadly all came to a crashing end in 2001 following a court decision that precluded the use of race in student school assignments [50, 51]. From 1980 to 2000, the index of dissimilarity in the Charlotte MSA dropped from 0.68 to 0.56, but it has remained at that level in the two decades since schools were resegregated.

## Limitations

This paper is subject to several important limitations. First, this is an ecological analysis and is potentially subject to confounding. One potential confounder is the possibility that wealthier people—who are more likely to be healthier—might tend to move into less segregated areas. In other words, there is a possibility for “reverse causation,” meaning that the health status of individuals may influence changes in segregation levels, rather than changes in segregation affecting health outcomes. However, this limitation is mitigated by our use of the MSA as the geographical unit of analysis. Overall rates of death in large metropolitan areas are unlikely to be explained by movements of individuals. It was for this reason that we chose to only include MSAs with a population of at least 100,000 back in 1980, including at least 5000 Black residents.

Second, in order to limit our analysis to large metropolitan areas with consistently defined populations throughout the period 1980–2020 and with a sizable enough Black population to derive stable mortality rate estimates, we necessarily excluded a number of MSAs. For example, our sample consists of 220 MSAs, while there are currently 404 MSAs (as defined in 2020). This may limit the generalizability of our findings. Nevertheless, the MSAs in our sample account for 44.8% of the non-Hispanic White US population in 2010 and 74% of the non-Hispanic Black population.

Third, we examined a specific subset of causes of death that are known to be related to racial disparities. Our results should not be extrapolated to other causes of death. Similarly, we examined death rates for people under age 65 because the higher longevity of the White population eventually reduces overall, non-age-adjusted racial disparities. Our results should not be used to draw conclusions about all-age mortality rates.

Finally, we only examined Black-White racial disparities because these are the largest disparities in major diseases across MSAs and provide the greatest statistical power to detect a relationship with racial segregation if one exists. Future research should examine health disparities among other racial/ethnic groups.

## Conclusion

The findings of this paper provide strong new evidence not only that racial residential segregation is significantly associated with Black-White racial health disparities, but that changes in segregation over time are also related to the level of and trends in these disparities. If further research confirms these findings, then combating racial segregation may be shown to be an effective strategy for reducing racial health disparities.

## Supplementary Information

Below is the link to the electronic supplementary material.

Supplementary file1 (DOCX 71 KB)

## Data Availability

The database of mortality rate ratios and segregation indices produced in this research project is available upon request from the lead author.

## Code Availability

Code used to run the trajectory analysis is available upon request from the lead author.

## Abbreviations

*CDC*: Centers for Disease Control and Prevention
*ICPSR*: Inter-university Consortium for Political and Social Research
*MSA*: Metropolitan statistical area
