# Supplementary material for: Association Between Changes in Racial Residential Segregation and Trends in Racial Disparities in Early Mortality in 220 Metropolitan Areas, 2001–2018
Source: J Racial Ethn Health Disparities. 2023 Oct 19;11(6):3782–93. doi: 10.1007/s40615-023-01830-z (PMC11564216; doi:10.1007/s40615-023-01830-z)
Supplement: Supplementary file 1 — Supplementary file1 (DOCX 71 KB) [file 40615_2023_1830_MOESM1_ESM.docx]

**Appendix Table 1.** Average Black-White Mortality Rate Ratios, Changes in Rate Ratios, Levels of Racial Residential Segregation as Measured by the Index of Dissimilarity, Changes in Segregation, and Trajectory Groups—220 Metropolitan Statistical Areas, 2001-2018

| **MSA Name** | **Counties in MSA** | **Average Black-White Mortality Rate Ratio, 2001-2018** | **Black-White Mortality Rate Ratio, 2001** | **Black-White Mortality Rate Ratio, 2018** | **Percent Change in Black-White Mortality Rate Ratio, 2001-2018** | **Index of Dissimilarity, 1980** | **Index of Dissimilarity, 2000** | **Change in Index of Dissimilarity, 1980-2000** | **Trajectory Group** |
| --- | --- | --- | --- | --- | --- | --- | --- | --- | --- |
| San Jose-Sunnyvale-Santa Clara, CA | Santa Clara County, CA | 4.07 | 3.31 | 4.52 | 26.7 | 0.49 | 0.62 | 0.13 | 3 |
| Washington-Arlington-Alexandria, DC-VA-MD | District of Columbia, DC; Prince George's County, MD; Fairfax County, VA | 3.35 | 3.24 | 3.75 | 13.7 | 0.74 | 0.73 | -0.01 | 3 |
| Athens-Clarke County, GA | Clarke County, GA | 3.19 | 3.15 | 3.13 | -0.7 | 0.55 | 0.42 | -0.13 | 3 |
| New Brunswick-Lakewood, NJ | Middlesex County, NJ; Monmouth County, NJ; Ocean County, NJ; Somerset County, NJ | 2.92 | 3.26 | 2.82 | -15.5 | 0.66 | 0.80 | 0.14 | 3 |
| Chicago-Naperville-Evanston, IL | Cook County, IL; DuPage County, IL | 2.87 | 2.67 | 3.53 | 24.4 | 0.90 | 0.83 | -0.07 | 3 |
| Oakland-Berkeley-Livermore, CA | Alameda County, CA; Contra Costa County, CA | 2.81 | 2.65 | 2.76 | 3.9 | 0.74 | 0.64 | -0.10 | 3 |
| Wilmington, NC | New Hanover County, NC | 2.67 | 2.61 | 2.76 | 5.7 | 0.65 | 0.53 | -0.12 | 3 |
| Charlottesville, VA | Albemarle County, VA; Charlottesville city, VA | 2.56 | 2.67 | 2.93 | 9.1 | 0.45 | 0.36 | -0.10 | 3 |
| Salinas, CA | Monterey County, CA | 2.55 | 2.37 | 3.15 | 24.6 | 0.67 | 0.72 | 0.05 | 3 |
| Trenton-Princeton, NJ | Mercer County, NJ | 2.48 | 2.34 | 2.47 | 5.3 | 0.71 | 0.64 | -0.07 | 3 |
| Newark, NJ | Essex County, NJ; Morris County, NJ; Union County, NJ | 2.44 | 2.08 | 2.94 | 29.2 | 0.82 | 0.84 | 0.02 | 3 |
| Durham-Chapel Hill, NC | Chatham County, NC; Durham County, NC; Orange County, NC | 2.42 | 2.44 | 2.48 | 1.7 | 0.54 | 0.49 | -0.05 | 3 |
| Miami-Miami Beach-Kendall, FL | Miami-Dade County, FL | 2.37 | 2.38 | 2.55 | 6.8 | 0.80 | 0.73 | -0.08 | 3 |
| Los Angeles-Long Beach-Glendale, CA | Los Angeles County, CA | 2.36 | 2.24 | 2.58 | 13.2 | 0.81 | 0.69 | -0.12 | 3 |
| Baton Rouge, LA | Ascension Parish, LA; East Baton Rouge Parish, LA; Livingston Parish, LA | 2.36 | 2.86 | 2.12 | -34.4 | 0.72 | 0.67 | -0.05 | 3 |
| Kansas City, MO-KS | Johnson County, KS; Jackson County, MO | 2.35 | 2.40 | 2.70 | 11.4 | 0.81 | 0.72 | -0.09 | 3 |
| Baltimore-Columbia-Towson, MD | Anne Arundel County, MD; Baltimore County, MD; Howard County, MD; Baltimore city, MD | 2.32 | 2.87 | 2.26 | -26.9 | 0.75 | 0.68 | -0.07 | 3 |
| New York, NY | Bronx County, NY; Kings County, NY; New York County, NY; Queens County, NY | 2.30 | 2.28 | 2.30 | 0.9 | 0.83 | 0.71 | -0.12 | 3 |
| Saginaw, MI | Saginaw County, MI | 2.29 | 1.98 | 2.11 | 6.4 | 0.84 | 0.73 | -0.10 | 3 |
| Omaha, NE | Douglas County, NE; Sarpy County, NE | 2.29 | 2.37 | 2.27 | -4.3 | 0.74 | 0.64 | -0.09 | 3 |
| Pittsburgh, PA | Allegheny County, PA; Westmoreland County, PA | 2.26 | 2.28 | 2.14 | -6.4 | 0.76 | 0.70 | -0.06 | 3 |
| Detroit-Dearborn-Livonia, MI | Wayne County, MI | 2.20 | 2.25 | 2.23 | -1.0 | 0.83 | 0.86 | 0.03 | 3 |
| Atlanta-Sandy Springs-Alpharetta, GA | Cobb County, GA; DeKalb County, GA; Fulton County, GA; Gwinnett County, GA | 2.18 | 2.39 | 2.16 | -10.5 | 0.80 | 0.69 | -0.12 | 3 |
| Lubbock, TX | Lubbock County, TX | 2.18 | 2.00 | 2.17 | 7.7 | 0.69 | 0.55 | -0.14 | 3 |
| Buffalo-Cheektowaga, NY | Erie County, NY; Niagara County, NY | 2.18 | 2.21 | 2.12 | -3.9 | 0.80 | 0.78 | -0.02 | 3 |
| Atlantic City-Hammonton, NJ | Atlantic County, NJ | 2.14 | 2.25 | 2.07 | -9.0 | 0.74 | 0.63 | -0.11 | 2 |
| Charlotte-Concord-Gastonia, NC-SC | Mecklenburg County, NC; York County, SC | 2.14 | 2.62 | 2.12 | -23.3 | 0.68 | 0.56 | -0.12 | 2 |
| Bridgeport-Stamford-Norwalk, CT | Fairfield County, CT | 2.13 | 2.22 | 2.09 | -6.4 | 0.70 | 0.70 | -0.01 | 2 |
| Raleigh-Cary, NC | Johnston County, NC; Wake County, NC | 2.11 | 2.40 | 2.10 | -14.2 | 0.51 | 0.41 | -0.10 | 2 |
| Richmond, VA | Chesterfield County, VA; Henrico County, VA; Richmond city, VA | 2.11 | 2.29 | 2.12 | -8.0 | 0.69 | 0.59 | -0.10 | 2 |
| Austin-Round Rock-Georgetown, TX | Travis County, TX; Williamson County, TX | 2.09 | 2.22 | 2.01 | -10.7 | 0.66 | 0.54 | -0.12 | 2 |
| Philadelphia, PA | Delaware County, PA; Philadelphia County, PA | 2.09 | 2.13 | 2.19 | 2.8 | 0.84 | 0.78 | -0.06 | 2 |
| Santa Maria-Santa Barbara, CA | Santa Barbara County, CA | 2.08 | 2.18 | 2.51 | 13.0 | 0.45 | 0.57 | 0.12 | 2 |
| Ann Arbor, MI | Washtenaw County, MI | 2.06 | 2.17 | 2.41 | 10.2 | 0.49 | 0.53 | 0.03 | 2 |
| Charleston-North Charleston, SC | Berkeley County, SC; Charleston County, SC; Dorchester County, SC | 2.05 | 2.00 | 2.07 | 3.3 | 0.57 | 0.44 | -0.13 | 2 |
| Rochester, NY | Monroe County, NY | 2.02 | 2.19 | 2.28 | 3.9 | 0.71 | 0.70 | -0.01 | 2 |
| Madison, WI | Dane County, WI | 2.01 | 1.61 | 2.24 | 28.2 | 0.47 | 0.46 | -0.01 | 2 |
| Lake County, IL | Lake County, IL | 2.00 | 1.83 | 2.40 | 23.8 | 0.74 | 0.70 | -0.04 | 2 |
| Gary, IN | Lake County, IN | 1.98 | 2.29 | 1.84 | -24.6 | 0.89 | 0.83 | -0.06 | 2 |
| Milwaukee-Waukesha, WI | Milwaukee County, WI | 1.96 | 1.76 | 2.30 | 23.3 | 0.81 | 0.78 | -0.03 | 2 |
| Champaign-Urbana, IL | Champaign County, IL | 1.96 | 1.99 | 2.30 | 13.7 | 0.51 | 0.46 | -0.06 | 2 |
| Memphis, TN-MS | DeSoto County, MS; Shelby County, TN | 1.95 | 2.17 | 2.00 | -8.5 | 0.73 | 0.71 | -0.02 | 2 |
| Virginia Beach-Norfolk-Newport News, VA | Chesapeake city, VA; Newport News city, VA; Norfolk city, VA; Virginia Beach city, VA | 1.94 | 1.93 | 1.95 | 0.9 | 0.61 | 0.45 | -0.16 | 2 |
| Birmingham-Hoover, AL | Jefferson County, AL; Shelby County, AL | 1.94 | 2.21 | 1.96 | -12.4 | 0.76 | 0.70 | -0.06 | 2 |
| Dallas-Plano-Irving, TX | Collin County, TX; Dallas County, TX; Denton County, TX | 1.93 | 2.12 | 1.95 | -8.6 | 0.79 | 0.60 | -0.19 | 2 |
| Columbia, MO | Boone County, MO | 1.92 | 1.76 | 2.37 | 26.0 | 0.46 | 0.39 | -0.07 | 2 |
| Nashville-Davidson--Murfreesboro--Franklin, TN | Davidson County, TN; Rutherford County, TN; Williamson County, TN | 1.90 | 2.16 | 1.89 | -14.6 | 0.68 | 0.55 | -0.13 | 2 |
| Fort Wayne, IN | Allen County, IN | 1.90 | 2.01 | 1.83 | -10.1 | 0.74 | 0.69 | -0.05 | 2 |
| Cleveland-Elyria, OH | Cuyahoga County, OH; Lorain County, OH | 1.89 | 1.85 | 2.03 | 8.6 | 0.85 | 0.77 | -0.08 | 2 |
| Shreveport-Bossier City, LA | Bossier Parish, LA; Caddo Parish, LA | 1.88 | 2.06 | 1.96 | -4.9 | 0.67 | 0.53 | -0.14 | 2 |
| Waterloo-Cedar Falls, IA | Black Hawk County, IA | 1.88 | 2.35 | 1.82 | -28.9 | 0.75 | 0.67 | -0.08 | 2 |
| Gainesville, FL | Alachua County, FL | 1.88 | 1.93 | 1.75 | -10.5 | 0.52 | 0.42 | -0.11 | 2 |
| Youngstown-Warren-Boardman, OH-PA | Mahoning County, OH; Trumbull County, OH; Mercer County, PA | 1.87 | 1.98 | 1.79 | -10.3 | 0.78 | 0.73 | -0.06 | 2 |
| El Paso, TX | El Paso County, TX | 1.87 | 2.29 | 2.29 | -0.1 | 0.43 | 0.53 | 0.10 | 2 |
| Cincinnati, OH | Butler County, OH; Hamilton County, OH | 1.86 | 1.88 | 1.82 | -3.3 | 0.77 | 0.70 | -0.07 | 2 |
| Harrisburg-Carlisle, PA | Dauphin County, PA | 1.85 | 1.82 | 1.80 | -1.3 | 0.69 | 0.64 | -0.04 | 2 |
| Wichita Falls, TX | Wichita County, TX | 1.85 | 1.97 | 1.67 | -18.2 | 0.71 | 0.58 | -0.12 | 2 |
| Grand Rapids-Kentwood, MI | Kent County, MI | 1.85 | 1.82 | 1.89 | 3.7 | 0.74 | 0.63 | -0.11 | 2 |
| Racine, WI | Racine County, WI | 1.85 | 1.55 | 1.99 | 22.2 | 0.69 | 0.56 | -0.13 | 2 |
| Knoxville, TN | Knox County, TN | 1.84 | 2.18 | 2.01 | -8.3 | 0.68 | 0.56 | -0.12 | 2 |
| Topeka, KS | Shawnee County, KS | 1.83 | 1.65 | 1.89 | 13.0 | 0.54 | 0.48 | -0.06 | 2 |
| Jackson, MS | Hinds County, MS; Madison County, MS; Rankin County, MS | 1.83 | 1.81 | 1.84 | 1.8 | 0.71 | 0.62 | -0.10 | 2 |
| Flint, MI | Genesee County, MI | 1.83 | 1.91 | 1.75 | -9.6 | 0.85 | 0.78 | -0.07 | 2 |
| Waco, TX | McLennan County, TX | 1.83 | 2.06 | 1.86 | -10.8 | 0.66 | 0.51 | -0.14 | 2 |
| Asheville, NC | Buncombe County, NC | 1.82 | 2.02 | 2.08 | 2.7 | 0.66 | 0.57 | -0.09 | 2 |
| Chattanooga, TN | Hamilton County, TN | 1.80 | 2.06 | 1.64 | -25.4 | 0.73 | 0.68 | -0.05 | 2 |
| Weirton-Steubenville, OH | Jefferson County, OH | 1.79 | 1.50 | 1.85 | 19.2 | 0.67 | 0.62 | -0.06 | 2 |
| Port St. Lucie, FL | Martin County, FL; St. Lucie County, FL | 1.76 | 1.68 | 1.90 | 11.5 | 0.84 | 0.55 | -0.29 | 2 |
| Houma-Thibodaux, LA | Lafourche Parish, LA; Terrebonne Parish, LA | 1.76 | 1.89 | 1.74 | -8.7 | 0.62 | 0.46 | -0.16 | 2 |
| Houston-The Woodlands-Sugar Land, TX | Fort Bend County, TX; Harris County, TX | 1.76 | 1.87 | 1.82 | -2.4 | 0.77 | 0.67 | -0.10 | 2 |
| Lexington-Fayette, KY | Fayette County, KY | 1.76 | 1.93 | 1.70 | -13.2 | 0.66 | 0.48 | -0.18 | 2 |
| Seattle-Bellevue-Kent, WA | King County, WA | 1.76 | 2.08 | 1.60 | -30.3 | 0.68 | 0.56 | -0.12 | 2 |
| Fresno, CA | Fresno County, CA | 1.76 | 1.68 | 1.77 | 5.3 | 0.64 | 0.53 | -0.11 | 2 |
| Denver-Aurora-Lakewood, CO | Adams County, CO; Arapahoe County, CO; Denver County, CO | 1.75 | 1.78 | 1.93 | 7.8 | 0.66 | 0.58 | -0.09 | 2 |
| Lafayette, LA | Acadia Parish, LA; Iberia Parish, LA; Lafayette Parish, LA; Vermilion Parish, LA | 1.75 | 1.88 | 1.80 | -4.5 | 0.56 | 0.50 | -0.07 | 2 |
| Spartanburg, SC | Spartanburg County, SC | 1.74 | 1.86 | 1.82 | -1.8 | 0.48 | 0.60 | 0.12 | 2 |
| Dayton-Kettering, OH | Greene County, OH; Montgomery County, OH | 1.72 | 1.65 | 1.67 | 0.8 | 0.80 | 0.73 | -0.07 | 2 |
| Camden, NJ | Burlington County, NJ; Camden County, NJ; Gloucester County, NJ | 1.71 | 1.88 | 1.61 | -16.9 | 0.60 | 0.57 | -0.03 | 2 |
| Montgomery, AL | Autauga County, AL; Elmore County, AL; Montgomery County, AL | 1.71 | 1.77 | 1.69 | -4.6 | 0.62 | 0.53 | -0.09 | 2 |
| Wilmington, DE-MD | New Castle County, DE; Cecil County, MD | 1.71 | 2.12 | 1.43 | -48.7 | 0.64 | 0.53 | -0.11 | 2 |
| Syracuse, NY | Onondaga County, NY | 1.71 | 2.04 | 1.74 | -17.4 | 0.73 | 0.69 | -0.04 | 2 |
| Reading, PA | Berks County, PA | 1.71 | 1.90 | 1.19 | -59.7 | 0.66 | 0.59 | -0.06 | 2 |
| South Bend-Mishawaka, IN-MI | St. Joseph County, IN; Cass County, MI | 1.68 | 1.83 | 1.73 | -5.6 | 0.66 | 0.58 | -0.08 | 2 |
| Alexandria, LA | Rapides Parish, LA | 1.68 | 1.55 | 1.69 | 8.1 | 0.69 | 0.60 | -0.09 | 2 |
| Columbia, SC | Lexington County, SC; Richland County, SC | 1.68 | 1.85 | 1.59 | -16.5 | 0.59 | 0.51 | -0.08 | 2 |
| Mobile, AL | Mobile County, AL | 1.68 | 1.74 | 1.43 | -21.6 | 0.74 | 0.64 | -0.10 | 2 |
| York-Hanover, PA | York County, PA | 1.67 | 2.28 | 1.68 | -36.1 | 0.76 | 0.70 | -0.06 | 2 |
| Jacksonville, FL | Clay County, FL; Duval County, FL; St. Johns County, FL | 1.67 | 1.75 | 1.55 | -12.9 | 0.70 | 0.54 | -0.16 | 2 |
| San Francisco-San Mateo-Redwood City, CA | San Francisco County, CA; San Mateo County, CA | 1.67 | 1.48 | 1.70 | 13.1 | 0.66 | 0.56 | -0.11 | 2 |
| Canton-Massillon, OH | Stark County, OH | 1.67 | 2.05 | 1.52 | -34.3 | 0.69 | 0.58 | -0.11 | 2 |
| Tallahassee, FL | Leon County, FL | 1.66 | 1.75 | 1.48 | -18.3 | 0.55 | 0.41 | -0.13 | 2 |
| Albany-Schenectady-Troy, NY | Albany County, NY; Rensselaer County, NY; Schenectady County, NY | 1.66 | 1.84 | 1.61 | -13.9 | 0.64 | 0.61 | -0.03 | 2 |
| New Orleans-Metairie, LA | Jefferson Parish, LA; Orleans Parish, LA; St. Tammany Parish, LA | 1.65 | 1.94 | 1.47 | -31.5 | 0.72 | 0.67 | -0.05 | 2 |
| Longview, TX | Gregg County, TX; Harrison County, TX | 1.65 | 1.72 | 1.91 | 10.2 | 0.52 | 0.46 | -0.06 | 2 |
| Tulsa, OK | Tulsa County, OK | 1.65 | 1.63 | 1.65 | 1.5 | 0.81 | 0.57 | -0.24 | 2 |
| Warren-Troy-Farmington Hills, MI | Macomb County, MI; Oakland County, MI | 1.64 | 1.61 | 1.69 | 5.3 | 0.81 | 0.70 | -0.11 | 2 |
| Merced, CA | Merced County, CA | 1.64 | 1.72 | 1.73 | 0.1 | 0.46 | 0.34 | -0.12 | 2 |
| Akron, OH | Portage County, OH; Summit County, OH | 1.64 | 1.64 | 1.61 | -1.8 | 0.75 | 0.66 | -0.09 | 2 |
| Lansing-East Lansing, MI | Ingham County, MI | 1.64 | 1.71 | 1.53 | -11.2 | 0.50 | 0.51 | 0.01 | 2 |
| St. Louis, MO | St. Louis County, MO; St. Louis city, MO | 1.63 | 1.59 | 1.39 | -14.3 | 0.80 | 0.64 | -0.16 | 2 |
| Columbus, OH | Franklin County, OH | 1.63 | 1.77 | 1.54 | -15.1 | 0.72 | 0.60 | -0.12 | 2 |
| Hartford-East Hartford-Middletown, CT | Hartford County, CT; Middlesex County, CT | 1.62 | 1.87 | 1.51 | -24.0 | 0.72 | 0.66 | -0.05 | 2 |
| Utica-Rome, NY | Oneida County, NY | 1.62 | 1.61 | 1.42 | -13.8 | 0.62 | 0.62 | 0.00 | 2 |
| Greenville-Anderson, SC | Anderson County, SC; Greenville County, SC; Pickens County, SC | 1.62 | 1.92 | 1.57 | -22.6 | 0.56 | 0.46 | -0.10 | 2 |
| Montgomery County-Bucks County-Chester County, PA | Bucks County, PA; Chester County, PA; Montgomery County, PA | 1.62 | 1.79 | 1.63 | -9.4 | 0.59 | 0.55 | -0.04 | 2 |
| Louisville/Jefferson County, KY | Jefferson County, KY | 1.62 | 1.64 | 1.64 | 0.2 | 0.76 | 0.65 | -0.11 | 2 |
| Toledo, OH | Lucas County, OH | 1.62 | 1.63 | 1.52 | -7.3 | 0.78 | 0.68 | -0.10 | 2 |
| West Palm Beach-Boca Raton-Boynton Beach, FL | Palm Beach County, FL | 1.62 | 1.88 | 1.39 | -34.6 | 0.84 | 0.66 | -0.18 | 2 |
| Winston-Salem, NC | Davidson County, NC; Forsyth County, NC | 1.61 | 1.97 | 1.47 | -34.0 | 0.73 | 0.61 | -0.12 | 2 |
| Kalamazoo-Portage, MI | Kalamazoo County, MI | 1.60 | 1.60 | 1.75 | 8.4 | 0.58 | 0.49 | -0.09 | 2 |
| Lancaster, PA | Lancaster County, PA | 1.60 | 1.42 | 1.33 | -6.6 | 0.69 | 0.60 | -0.08 | 2 |
| Muskegon, MI | Muskegon County, MI | 1.60 | 1.46 | 1.69 | 13.2 | 0.75 | 0.78 | 0.03 | 2 |
| Nassau County-Suffolk County, NY | Nassau County, NY; Suffolk County, NY | 1.60 | 1.82 | 1.60 | -13.5 | 0.78 | 0.75 | -0.03 | 2 |
| Lake Charles, LA | Calcasieu Parish, LA | 1.60 | 1.85 | 1.42 | -30.5 | 0.69 | 0.62 | -0.07 | 2 |
| North Port-Sarasota-Bradenton, FL | Manatee County, FL; Sarasota County, FL | 1.59 | 1.66 | 1.53 | -8.4 | 0.85 | 0.67 | -0.18 | 2 |
| San Rafael, CA | Marin County, CA | 1.59 | 1.62 | 1.61 | -1.0 | 0.52 | 0.41 | -0.11 | 2 |
| Kankakee, IL | Kankakee County, IL | 1.58 | 2.13 | 1.71 | -24.6 | 0.75 | 0.71 | -0.04 | 2 |
| Little Rock-North Little Rock-Conway, AR | Pulaski County, AR | 1.58 | 1.62 | 1.65 | 1.7 | 0.65 | 0.59 | -0.06 | 2 |
| Monroe, LA | Morehouse Parish, LA; Ouachita Parish, LA | 1.58 | 1.58 | 1.49 | -5.9 | 0.72 | 0.65 | -0.07 | 2 |
| Boston, MA | Norfolk County, MA; Plymouth County, MA; Suffolk County, MA | 1.57 | 1.73 | 1.48 | -16.9 | 0.80 | 0.73 | -0.07 | 2 |
| New Haven-Milford, CT | New Haven County, CT | 1.57 | 1.77 | 1.55 | -14.0 | 0.69 | 0.59 | -0.10 | 2 |
| Minneapolis-St. Paul-Bloomington, MN | Hennepin County, MN; Ramsey County, MN | 1.57 | 1.53 | 1.83 | 16.0 | 0.66 | 0.57 | -0.08 | 2 |
| Fayetteville, NC | Cumberland County, NC; Harnett County, NC | 1.57 | 1.68 | 1.41 | -19.3 | 0.39 | 0.33 | -0.06 | 2 |
| Stockton, CA | San Joaquin County, CA | 1.56 | 1.42 | 1.54 | 8.3 | 0.65 | 0.55 | -0.09 | 2 |
| Vallejo, CA | Solano County, CA | 1.56 | 1.46 | 1.59 | 8.4 | 0.41 | 0.43 | 0.02 | 2 |
| San Diego-Chula Vista-Carlsbad, CA | San Diego County, CA | 1.56 | 1.47 | 1.59 | 7.8 | 0.64 | 0.48 | -0.16 | 2 |
| Lima, OH | Allen County, OH | 1.55 | 1.65 | 1.70 | 3.3 | 0.64 | 0.55 | -0.09 | 2 |
| Lynchburg, VA | Amherst County, VA; Bedford County, VA; Campbell County, VA; Lynchburg city, VA | 1.55 | 1.63 | 1.58 | -3.0 | 0.43 | 0.39 | -0.04 | 2 |
| Portland, OR | Multnomah County, OR | 1.55 | 2.17 | 1.12 | -93.5 | 0.70 | 0.61 | -0.09 | 2 |
| Niles, MI | Berrien County, MI | 1.55 | 1.98 | 1.56 | -26.8 | 0.74 | 0.75 | 0.01 | 2 |
| Abilene, TX | Taylor County, TX | 1.55 | 1.81 | 1.15 | -58.3 | 0.50 | 0.40 | -0.10 | 2 |
| Springfield, OH | Clark County, OH | 1.54 | 1.54 | 1.56 | 1.3 | 0.68 | 0.68 | 0.00 | 2 |
| Corpus Christi, TX | Nueces County, TX | 1.54 | 1.99 | 1.35 | -47.7 | 0.72 | 0.48 | -0.24 | 2 |
| Erie, PA | Erie County, PA | 1.53 | 1.91 | 1.41 | -35.6 | 0.64 | 0.66 | 0.01 | 2 |
| Peoria, IL | Peoria County, IL | 1.53 | 1.40 | 1.76 | 20.5 | 0.61 | 0.58 | -0.02 | 2 |
| Sacramento-Roseville-Folsom, CA | Sacramento County, CA | 1.53 | 1.50 | 1.63 | 7.7 | 0.53 | 0.49 | -0.05 | 2 |
| Tuscaloosa, AL | Tuscaloosa County, AL | 1.52 | 1.42 | 1.54 | 8.1 | 0.52 | 0.53 | 0.01 | 2 |
| Poughkeepsie-Newburgh-Middletown, NY | Dutchess County, NY; Orange County, NY | 1.52 | 1.78 | 1.24 | -44.1 | 0.55 | 0.53 | -0.03 | 2 |
| Frederick-Gaithersburg-Rockville, MD | Frederick County, MD; Montgomery County, MD | 1.52 | 1.66 | 1.45 | -14.9 | 0.45 | 0.47 | 0.02 | 2 |
| Battle Creek, MI | Calhoun County, MI | 1.52 | 1.74 | 1.37 | -27.4 | 0.72 | 0.61 | -0.11 | 2 |
| Jacksonville, NC | Onslow County, NC | 1.51 | 1.59 | 1.63 | 2.2 | 0.34 | 0.26 | -0.08 | 1 |
| Phoenix-Mesa-Chandler, AZ | Maricopa County, AZ | 1.51 | 1.61 | 1.49 | -7.9 | 0.62 | 0.45 | -0.17 | 1 |
| Elgin, IL | Kane County, IL | 1.50 | 1.44 | 1.78 | 19.4 | 0.57 | 0.57 | 0.00 | 1 |
| Hattiesburg, MS | Covington County, MS; Forrest County, MS | 1.50 | 1.33 | 1.59 | 16.6 | 0.68 | 0.51 | -0.17 | 1 |
| Augusta-Richmond County, GA-SC | Columbia County, GA; Richmond County, GA; Aiken County, SC | 1.49 | 1.74 | 1.36 | -27.6 | 0.49 | 0.46 | -0.03 | 1 |
| Oxnard-Thousand Oaks-Ventura, CA | Ventura County, CA | 1.49 | 1.68 | 1.40 | -19.8 | 0.57 | 0.49 | -0.08 | 1 |
| Evansville, IN | Vanderburgh County, IN | 1.49 | 1.45 | 1.36 | -6.5 | 0.71 | 0.56 | -0.15 | 1 |
| Anaheim-Santa Ana-Irvine, CA | Orange County, CA | 1.48 | 1.41 | 1.53 | 7.8 | 0.46 | 0.38 | -0.08 | 1 |
| Macon-Bibb County, GA | Bibb County, GA; Jones County, GA; Monroe County, GA | 1.48 | 1.72 | 1.58 | -8.8 | 0.56 | 0.56 | 0.00 | 1 |
| Tyler, TX | Smith County, TX | 1.48 | 1.50 | 1.62 | 7.2 | 0.56 | 0.53 | -0.04 | 1 |
| Anchorage, AK | Anchorage Municipality, AK | 1.47 | 1.34 | 1.50 | 10.1 | 0.39 | 0.37 | -0.02 | 1 |
| Indianapolis-Carmel-Anderson, IN | Marion County, IN | 1.47 | 1.60 | 1.46 | -9.1 | 0.75 | 0.62 | -0.13 | 1 |
| Des Moines-West Des Moines, IA | Polk County, IA | 1.46 | 1.73 | 1.52 | -13.5 | 0.69 | 0.56 | -0.13 | 1 |
| Springfield, MA | Hampden County, MA | 1.45 | 1.78 | 1.44 | -23.0 | 0.74 | 0.57 | -0.17 | 1 |
| Columbus, GA-AL | Russell County, AL; Harris County, GA; Muscogee County, GA | 1.45 | 1.48 | 1.33 | -11.1 | 0.58 | 0.60 | 0.02 | 1 |
| Rocky Mount, NC | Edgecombe County, NC; Nash County, NC | 1.45 | 1.60 | 1.27 | -26.7 | 0.45 | 0.40 | -0.05 | 1 |
| Rockford, IL | Winnebago County, IL | 1.45 | 1.59 | 1.45 | -9.5 | 0.77 | 0.60 | -0.16 | 1 |
| Albuquerque, NM | Bernalillo County, NM | 1.44 | 1.37 | 1.55 | 11.7 | 0.41 | 0.34 | -0.07 | 1 |
| Jackson, MI | Jackson County, MI | 1.44 | 1.62 | 1.37 | -18.3 | 0.75 | 0.67 | -0.08 | 1 |
| Springfield, IL | Sangamon County, IL | 1.44 | 1.59 | 1.29 | -23.3 | 0.68 | 0.40 | -0.28 | 1 |
| Orlando-Kissimmee-Sanford, FL | Lake County, FL; Orange County, FL; Osceola County, FL; Seminole County, FL | 1.43 | 1.66 | 1.28 | -29.1 | 0.74 | 0.56 | -0.18 | 1 |
| Beaumont-Port Arthur, TX | Hardin County, TX; Jefferson County, TX; Orange County, TX | 1.42 | 1.45 | 1.45 | -0.5 | 0.76 | 0.70 | -0.06 | 1 |
| Dothan, AL | Geneva County, AL; Henry County, AL; Houston County, AL | 1.41 | 1.40 | 1.44 | 2.9 | 0.59 | 0.45 | -0.14 | 1 |
| Pensacola-Ferry Pass-Brent, FL | Escambia County, FL | 1.40 | 1.66 | 1.30 | -27.6 | 0.64 | 0.46 | -0.18 | 1 |
| Hickory-Lenoir-Morganton, NC | Burke County, NC; Caldwell County, NC; Catawba County, NC | 1.40 | 1.56 | 1.42 | -10.1 | 0.55 | 0.46 | -0.09 | 1 |
| Fort Worth-Arlington-Grapevine, TX | Tarrant County, TX | 1.39 | 1.73 | 1.28 | -34.7 | 0.78 | 0.58 | -0.20 | 1 |
| Odessa, TX | Ector County, TX | 1.38 | 1.56 | 0.75 | -108.8 | 0.78 | 0.48 | -0.30 | 1 |
| Salisbury, MD-DE | Sussex County, DE; Wicomico County, MD | 1.38 | 1.45 | 1.20 | -21.2 | 0.39 | 0.56 | 0.17 | 1 |
| Clarksville, TN-KY | Christian County, KY; Montgomery County, TN | 1.38 | 1.26 | 1.60 | 21.3 | 0.43 | 0.38 | -0.05 | 1 |
| Roanoke, VA | Franklin County, VA; Roanoke city, VA | 1.38 | 1.61 | 1.22 | -32.3 | 0.66 | 0.62 | -0.04 | 1 |
| Savannah, GA | Chatham County, GA; Effingham County, GA | 1.37 | 1.47 | 1.49 | 1.5 | 0.68 | 0.44 | -0.25 | 1 |
| Florence-Muscle Shoals, AL | Colbert County, AL; Lauderdale County, AL | 1.37 | 1.03 | 1.32 | 22.4 | 0.50 | 0.43 | -0.07 | 1 |
| Cape Coral-Fort Myers, FL | Lee County, FL | 1.37 | 1.71 | 1.16 | -47.6 | 0.89 | 0.69 | -0.19 | 1 |
| Colorado Springs, CO | El Paso County, CO | 1.36 | 1.44 | 1.36 | -5.8 | 0.45 | 0.42 | -0.03 | 1 |
| Davenport-Moline-Rock Island, IA-IL | Rock Island County, IL; Scott County, IA | 1.35 | 1.19 | 1.51 | 21.0 | 0.66 | 0.52 | -0.13 | 1 |
| Charleston, WV | Kanawha County, WV | 1.34 | 1.24 | 1.35 | 8.3 | 0.62 | 0.52 | -0.10 | 1 |
| San Antonio-New Braunfels, TX | Bexar County, TX | 1.33 | 1.56 | 1.21 | -29.5 | 0.62 | 0.36 | -0.26 | 1 |
| Greensboro-High Point, NC | Guilford County, NC; Randolph County, NC; Rockingham County, NC | 1.33 | 1.70 | 1.31 | -29.7 | 0.61 | 0.54 | -0.07 | 1 |
| Huntsville, AL | Limestone County, AL; Madison County, AL | 1.33 | 1.32 | 1.31 | -0.9 | 0.55 | 0.55 | -0.01 | 1 |
| Oklahoma City, OK | Oklahoma County, OK | 1.32 | 1.19 | 1.43 | 16.4 | 0.71 | 0.52 | -0.19 | 1 |
| Tampa-St. Petersburg-Clearwater, FL | Hillsborough County, FL; Pasco County, FL; Pinellas County, FL | 1.32 | 1.64 | 1.19 | -37.7 | 0.79 | 0.65 | -0.14 | 1 |
| Beckley, WV | Fayette County, WV; Raleigh County, WV | 1.31 | 1.31 | 1.11 | -18.0 | 0.55 | 0.44 | -0.11 | 1 |
| Las Vegas-Henderson-Paradise, NV | Clark County, NV | 1.31 | 1.43 | 1.36 | -5.4 | 0.63 | 0.40 | -0.23 | 1 |
| Decatur, IL | Macon County, IL | 1.30 | 1.40 | 1.19 | -17.3 | 0.64 | 0.54 | -0.10 | 1 |
| Mansfield, OH | Richland County, OH | 1.29 | 1.49 | 1.13 | -31.7 | 0.72 | 0.64 | -0.08 | 1 |
| Florence, SC | Florence County, SC | 1.28 | 1.52 | 1.29 | -17.3 | 0.54 | 0.42 | -0.12 | 1 |
| Allentown-Bethlehem-Easton, PA | Lehigh County, PA; Northampton County, PA | 1.26 | 1.39 | 1.08 | -28.8 | 0.58 | 0.53 | -0.05 | 1 |
| Fort Smith, AR | Sebastian County, AR | 1.26 | 1.25 | 1.18 | -6.3 | 0.67 | 0.55 | -0.12 | 1 |
| Palm Bay-Melbourne-Titusville, FL | Brevard County, FL | 1.26 | 1.58 | 1.17 | -35.5 | 0.69 | 0.49 | -0.20 | 1 |
| Huntington, WV | Cabell County, WV | 1.26 | 1.70 | 1.15 | -47.3 | 0.73 | 0.60 | -0.14 | 1 |
| Riverside-San Bernardino-Ontario, CA | Riverside County, CA; San Bernardino County, CA | 1.26 | 1.18 | 1.31 | 10.3 | 0.55 | 0.47 | -0.08 | 1 |
| Gadsden, AL | Etowah County, AL | 1.25 | 1.20 | 1.13 | -6.3 | 0.71 | 0.70 | -0.02 | 1 |
| Gulfport-Biloxi, MS | Harrison County, MS; Jackson County, MS | 1.25 | 1.25 | 1.27 | 2.0 | 0.57 | 0.50 | -0.07 | 1 |
| Texarkana, TX-AR | Miller County, AR; Bowie County, TX | 1.24 | 1.44 | 1.17 | -22.4 | 0.44 | 0.43 | -0.01 | 1 |
| Muncie, IN | Delaware County, IN | 1.23 | 1.23 | 1.20 | -2.3 | 0.72 | 0.55 | -0.18 | 1 |
| Bakersfield, CA | Kern County, CA | 1.21 | 1.15 | 1.33 | 13.3 | 0.65 | 0.52 | -0.13 | 1 |
| Tacoma-Lakewood, WA | Pierce County, WA | 1.21 | 1.16 | 1.35 | 14.7 | 0.52 | 0.47 | -0.05 | 1 |
| Vineland-Bridgeton, NJ | Cumberland County, NJ | 1.19 | 1.30 | 1.19 | -8.8 | 0.44 | 0.41 | -0.03 | 1 |
| Deltona-Daytona Beach-Ormond Beach, FL | Volusia County, FL | 1.16 | 1.33 | 0.96 | -38.3 | 0.79 | 0.58 | -0.21 | 1 |
| Providence-Warwick, RI-MA | Bristol County, MA; Providence County, RI | 1.16 | 1.57 | 0.88 | -77.9 | 0.70 | 0.56 | -0.13 | 1 |
| Fort Lauderdale-Pompano Beach-Sunrise, FL | Broward County, FL | 1.15 | 1.31 | 1.08 | -21.9 | 0.84 | 0.62 | -0.22 | 1 |
| Tucson, AZ | Pima County, AZ | 1.15 | 1.49 | 1.21 | -23.0 | 0.52 | 0.40 | -0.12 | 1 |
| Lakeland-Winter Haven, FL | Polk County, FL | 1.14 | 1.46 | 1.02 | -43.0 | 0.69 | 0.52 | -0.17 | 1 |
| Kingston, NY | Ulster County, NY | 1.14 | 1.63 | 0.99 | -65.1 | 0.49 | 0.44 | -0.05 | 1 |
| Albany, GA | Dougherty County, GA; Worth County, GA | 1.14 | 1.09 | 1.10 | 0.8 | 0.66 | 0.56 | -0.10 | 1 |
| Wichita, KS | Sedgwick County, KS | 1.13 | 1.12 | 1.20 | 6.8 | 0.73 | 0.52 | -0.21 | 1 |
| Anniston-Oxford, AL | Calhoun County, AL | 1.12 | 1.40 | 0.82 | -70.3 | 0.61 | 0.49 | -0.12 | 1 |
| Crestview-Fort Walton Beach-Destin, FL | Okaloosa County, FL | 1.12 | 1.12 | 1.10 | -2.1 | 0.51 | 0.30 | -0.21 | 1 |
| Norwich-New London, CT | New London County, CT | 1.09 | 1.35 | 1.07 | -26.1 | 0.52 | 0.54 | 0.03 | 1 |
| Terre Haute, IN | Vigo County, IN | 1.07 | 1.12 | 0.95 | -17.9 | 0.54 | 0.50 | -0.04 | 1 |
| Lawton, OK | Comanche County, OK | 1.06 | 0.86 | 1.28 | 32.7 | 0.40 | 0.32 | -0.09 | 1 |
| Killeen-Temple, TX | Bell County, TX; Coryell County, TX | 1.02 | 0.82 | 1.18 | 30.8 | 0.51 | 0.40 | -0.11 | 1 |
| Michigan City-La Porte, IN | LaPorte County, IN | 1.01 | 1.01 | 1.33 | 24.5 | 0.75 | 0.63 | -0.12 | 1 |
| Cambridge-Newton-Framingham, MA | Essex County, MA; Middlesex County, MA | 1.00 | 1.12 | 1.01 | -10.7 | 0.52 | 0.52 | 0.00 | 1 |
| Worcester, MA | Worcester County, MA | 1.00 | 1.25 | 0.97 | -29.1 | 0.54 | 0.53 | -0.02 | 1 |
| Elkhart-Goshen, IN | Elkhart County, IN | 0.99 | 0.88 | 0.98 | 10.4 | 0.74 | 0.36 | -0.38 | 1 |
| Ocala, FL | Marion County, FL | 0.95 | 1.17 | 0.88 | -32.2 | 0.62 | 0.50 | -0.12 | 1 |
| Amarillo, TX | Potter County, TX | 0.93 | 1.21 | 0.99 | -21.8 | 0.73 | 0.59 | -0.14 | 1 |
| Hagerstown, MD | Washington County, MD | 0.86 | 0.92 | 0.90 | -2.8 | 0.75 | 0.62 | -0.13 | 1 |
| Urban Honolulu, HI | Honolulu County, HI | 0.74 | 0.66 | 0.69 | 4.5 | 0.45 | 0.41 | -0.03 | 1 |
